# Multidisciplinary inpatient care for Parkinson’s disease: a single-centre cohort study on improvements in gait, overall motor function, and quality of life

**DOI:** 10.1186/s42466-025-00422-y

**Published:** 2025-09-02

**Authors:** Urs Kleinholdermann, Felicitas C. J. Mügge, Tiziano Carapezza, Lukas Decher, Lars Timmermann, David J. Pedrosa

**Affiliations:** 1 Department of Neurology, Philipps-University Marburg, Biegenstraße 10, 35032 Marburg, Germany; 2 Center of Mind, Brain and Behaviour, Philipps-University Marburg, Marburg, Germany

**Keywords:** Parkinson’s disease, Multidisciplinary care, Complex treatment, Wearables

## Abstract

**Background:**

Parkinson’s disease (PD) multimodal complex treatment (PD-MCT) is an inpatient therapeutic programme specifically designed for patients exhibiting parkinsonian symptoms. Established in Germany, this comprehensive approach addresses the multifaceted challenges associated with the management of PD, particularly in advanced stages or when complications such as motor fluctuations, dyskinesia, or non-motor symptoms become pronounced. The programme integrates pharmacological optimization, physiotherapy, occupational therapy, speech therapy, and psychological support, among other complementary therapies, to enhance patient outcomes holistically. Despite its availability for seventeen years, only seven studies evaluating the effectiveness of PD-MCT have been conducted. In this study we evaluated the effects of PD-MCT with a special focus on gait, hypothesizing an improvement after the treatment.

**Methods:**

In this single-centre cohort study at a German university hospital we included patients with PD diagnosed by the Movement Disorder Society (MDS) criteria, aged 18–85 years, legal capacity to consent and admitted for treatment with PD-MCT. We assessed changes in motor and non-motor symptoms using Wilcoxon’s signed rank test on pre/post measurements of part III of the motor part of the MDS Unified Parkinson’s Disease Rating Scale (MDS-UPDRS), the Parkinson’s Disease Questionnaire (PDQ-39) and the Timed Up and Go Test (TUG). As a particular emphasis was placed on gait analysis we objectively measured gait throughout the treatment period using advanced mobile sensor technology and analysed gait speed, stride length and lift height using linear mixed effects models.

**Results:**

In our sample of 43 PD patients we found significant improvements in MDS-UPRDRS part III (V = 679, *p* = 0.001), PDQ-39 (V = 770, *p* < 0.001) and TUG (V = 753.5, *p* < 0.001) values. as well as in the assessed gait parameters gait speed (t = 66.44, *p* < 0.001), stride length (t = 62.67, *p* < 0.001) and lift height (t = 28.16, *p* < 0.001).

**Conclusions:**

Our results underscore the added value of a multimodal inpatient approach, thereby supporting its role as a justified investment in the management of complex PD cases. This work contributes to the expanding body of evidence advocating for integrated, multidisciplinary care models in the treatment of neurodegenerative disorders.

**Trial registration:**

This study has not been registered.

**Supplementary Information:**

The online version contains supplementary material available at 10.1186/s42466-025-00422-y.

## Background

Parkinson’s disease (PD) is the second most common neurodegenerative disorder, with its prevalence rising rapidly mainly due to demographic shifts [1, 2]. The disease is characterised by a diverse array of symptoms. While motor symptoms such as bradykinesia, rigidity, and tremor are hallmark features, these are frequently accompanied by non-motor symptoms, including hyposmia, sleep disorders, affective disturbances, constipation, and autonomic dysregulation [3, 4].

Over the past several decades, the availability of symptomatic treatments for individuals with PD has expanded significantly [5, 6]. Dopaminergic replacement therapies, including dopamine agonists and levodopa, remain the cornerstone of treatment, targeting one of the key pathological mechanisms by restoring depleted dopamine levels in the substantia nigra [7]. Other pharmacological options, such as COMT inhibitors and monoamine oxidase inhibitors, may be considered to enhance dopamine availability in the synaptic cleft [8]. Furthermore, advancements in pharmacokinetic formulations facilitate more consistent dopaminergic stimulation throughout the day, thereby improving symptom control. In addition to dopaminergic treatments, invasive therapies such as Deep Brain Stimulation (DBS) have emerged as valuable long-term options that address both motor and certain non-motor symptoms [9–11]. Despite the growing array of treatment options, the complexity of managing PD exacerbates as the disease progresses. Personalised therapeutic strategies are often necessary, given the variability in symptom presentation and disease trajectory among patients.

As PD progresses, the burden of symptoms intensifies, impairing daily functioning. Key indicators of advanced PD, include falls, delusions, hallucinations, cognitive decline, motor fluctuations, and levodopa-induced dyskinesias [12], complications which contribute to increased frailty and mortality [13].

Comorbidities further complicate the management of PD, constraining treatment options due to potential drug interactions and contraindications [3]. Thus, specialised care for PD is essential. In principle, outpatient care remains the standard and preferred setting for developing personalised therapeutic strategies [14]. Some circumstances in PD, like crisis events, motor fluctuations as well as the management of DBS treatment and non-motor symptoms, however, can make inpatient care necessary [15]. For these indications, among others,

the German healthcare system provides a specialised intervention known as Parkinson’s Multimodal Complex Treatment (PD-MCT) [16] which is recommended by the German guidelines for PD [17]. PD-MCT is a comprehensive treatment programme that most often spans a duration of fourteen days [18] and is administered by an interdisciplinary team comprising professionals from diverse fields. This team includes physiotherapists, occupational therapists, speech and language therapists, music therapists, neuropsychologists, specialised nurses, and movement disorders specialists. At the onset of treatment, specific objectives are established for each patient on an individualised basis, with progress monitored through weekly interdisciplinary meetings. Health insurance providers acknowledge the elevated costs associated with PD-MCT and offer reimbursement for this treatment. Although the advantages of PD-MCT are readily apparent, empirical evidence validating its effectiveness in several observational studies has only recently begun to emerge. Research conducted by Heimrich and Prell indicates that PD-MCT leads to improvements in motor function [19]. Additionally, Hartelt et al. reported that the motor enhancements resulting from PD-MCT persist for several weeks following hospital discharge [20]. Wagner et al., however, reported that several quality of life-related parameters deteriorated again after initial improvement from PD-MCT over a period of nine months [21]. In a study by Ziegler et al., which involved a substantial sample of 591 patients, an enhancement in activities of daily living (ADL) was identified, as evaluated by the MDS-UPDRS part II [22]. Predictors of ADL improvements as well as improvements in gait were described by Oppermann et al. [23]. Furthermore, Scherbaum et al. discovered not only improvements in motor symptoms but also more favourable scores on depression and quality of life assessments [24]. Recent reviews corroborate the beneficial effects of PD-MCT [15, 25] or the broader concept of multidisciplinary rehabilitation [26] on both motor and non-motor symptoms. Also a review on multidisciplinary PD care in in- as well as outpatient settings confirmed positive results e.g. in QoL, motor scores, and non-motor symptoms in several randomised controlled trials [27].

The present study aimed to evaluate the effectiveness of PD-MCT utilising standard clinical rating scales and questionnaires to assess motor function and overall well-being at both admission and discharge. For the first time, we integrated mobile electronic sensors to continuously monitor gait throughout the treatment period. This method capitalises on advancements in sensor technology, facilitating a more comprehensive evaluation of motor function, building upon prior research utilising a simplified two-sensor configuration [28]. We hypothesised that clinical rating scale values and mobile sensor measurements would show an improvement over the course of PD-MCT. Furthermore, we conducted exploratory analyses in order to identify factors influencing PD-MCT outcomes which could guide and facilitate future research.

## Methods

The study was conducted as a single-centre cohort study. It adhered to the Declaration of Helsinki and received approval from the Research Ethics Committee of the Philipps University, Germany (reference number 86/21). Written informed consent was obtained from all participants. The study has not been registered in a trial registry.

### Sample

Participants were patients diagnosed with PD based on the Movement Disorders Society criteria [29], who were admitted for at least 14 days of PD-MCT, were aged 18–85 years, and had legal capacity to consent. Exclusion criteria for this study included atypical parkinsonism, lack of legal capacity, pregnancy, severe psychiatric or neurological conditions or sensory impairments affecting participation. Initially, patients using walking aids or with multiple recent falls were excluded, but this criterion was revised because of too many exclusions. Patients were recruited from the University Hospital of Marburg over a 20-month period beginning in January 2021. Based on power calculations conducted before study begin we aimed at including 50 participants while assuming a drop-out rate of 10%. Detailed sample characteristics are found in Table 1.

### Procedure

All patients participated in a comprehensive PD-MCT programme, which encompassed a broad spectrum of therapeutic interventions tailored to address the complex needs of individuals with PD. This programme offered at least 7.5 h of a customised combination of speech therapy, occupational therapy, physiotherapy, music therapy, and specialised nursing care in consultation with a movement disorders specialist. The therapeutic regimen was customised for each patient, ensuring alignment with their specific needs and preferences. Additionally, medication regimens were regularly reviewed and optimised during daily consultations. For patients with deep brain stimulation (DBS), device settings were evaluated and adjusted as necessary to maximize therapeutic benefit.

**Fig. 1:**
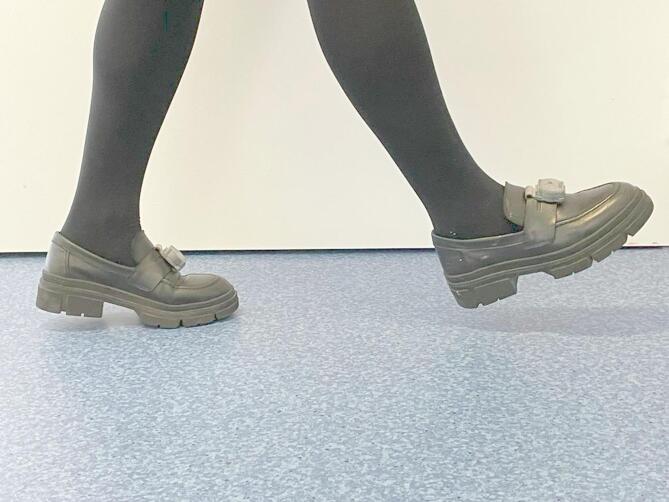
Gait sensors affixed to both shoes of a person in the position used during measurements (reproduced from [30])

At the onset of PD-MCT, patients were equipped with the Mobile GaitLab system (Mobile (Portabiles HealthCare Technologies GmbH). The system consists of two compact, portable devices, each containing an inertial measurement unit (IMU) sensor, designed to be affixed to the shoes (see Fig. 1) as well as a smartphone application. Patients were instructed to independently attach the sensors and use the smartphone to start and stop the measurements daily, promoting an active role in their treatment process.

To assess the programme’s impact on motor and non-motor symptoms, as well as overall patient satisfaction, participants completed standardised questionnaires and underwent comprehensive clinical evaluations at the first (or in some cases the second) and at the last day of the treatment period. This dual-assessment approach ensured a thorough evaluation of treatment outcomes, encompassing both subjective patient experiences and objective clinical measures.

### Measures

The impact of PD-MCT was evaluated using a combination of self-reported questionnaires, clinical assessments, and objective sensor-based measurements. Health-related quality of life was assessed pre- and post-treatment using the Parkinson’s Disease Questionnaire (PDQ-39) [31], which captures eight critical domains: mobility, daily activities, emotional well-being, stigma, social support, cognition, communication, and bodily discomfort. Additionally, at the end of the treatment we assessed overall satisfaction with PD-MCT and subjective improvement in four domains we deemed to be highly relevant to PD patients: gait, agility, daily living and tremor. This was done using a custom-designed five-item self-rating scale, with responses ranging from zero (does not apply) to four (fully applies) points.

Mobility was evaluated through two established clinical assessments: the Timed Up and Go (TUG) test [32] and part III of the MDS-UPDRS scale [33], which measures motor symptoms. To complement these clinical evaluations, gait was objectively assessed using the Mobile GaitLab system (Portabiles HealthCare Technologies GmbH), which employs IMU sensors to provide precise measurements. Key gait parameters, including speed, stride length, and maximum foot lift, were extracted using dynamic time warping techniques [30, 34, 35]. These parameters were analyzed as primary indicators of gait quality, providing a comprehensive view of the programme’s impact on motor function [28].

### Analyses

Descriptive statistics were calculated for pre- and post-treatment measurements derived from the PDQ-39 data to assess the impact of PD-MCT on health-related quality of life. The differences between these measurements were evaluated to quantify the treatment effect, with statistical significance determined using Wilcoxon’s signed rank test. For the custom-designed short questionnaire, median response values were computed across the sample to summarise overall feedback.

Clinical mobility metrics, including TUG and MDS-UPDRS part III scores, were also compared pre- and post-treatment using Wilcoxon’s signed rank test to identify significant changes. Gait parameters obtained from mobile sensor measurements were analysed using linear mixed models. This statistical approach allowed us to account for repeated measurements within subjects and to investigate the influence of medication adjustments on gait changes throughout the treatment period.

After completing these planned comparisons, exploratory analyses were conducted, including correlational studies and group comparisons, guided by visual inspection of the data to identify factors influencing the results. Improvements in gait parameters were further analysed through individual linear regression models to examine potential predictors of change.

All statistical analyses were performed using R (version 3.4.4) [36].

## Results

### Participants

A total of 45 patients (21 female) were recruited from the University Hospital of Marburg, with 43 completing the study over a 20-month period beginning in January 2021. Due to falls, we initially excluded 12 persons (cf. Methods section). After inclusion, one subject was excluded from the gait analysis due to a leg injury and three more because of technical issues with sensor recordings. One patient prematurely discontinued treatment after ten days but his data were kept for analysis. The average participant age was 65 years, with a median Hoehn & Yahr score of 2 (IQR: 1). Detailed sample characteristics are found in Table 1.

**Table 1:** Sample characteristics

*N*	43
age *mean (sd)*	65.33 (8.62)
‍sex (male / female)	22 / 21
Hoehn & Yahr stage *median (IQR)*	2 (1)
H/Y 1	3
H/Y 2	24
H/Y 3	14
H/Y 4	2
time since PD diagnosis in years *mean (sd)*	10.42 (7.04)
treatment duration in days *mean (sd)*	15.21 (1.1)
patients treated with DBS	18
patients using walking aids	
- walker	6
- stick	3
- crutches	1

### Main results

#### PDQ39

**Fig. 2:**
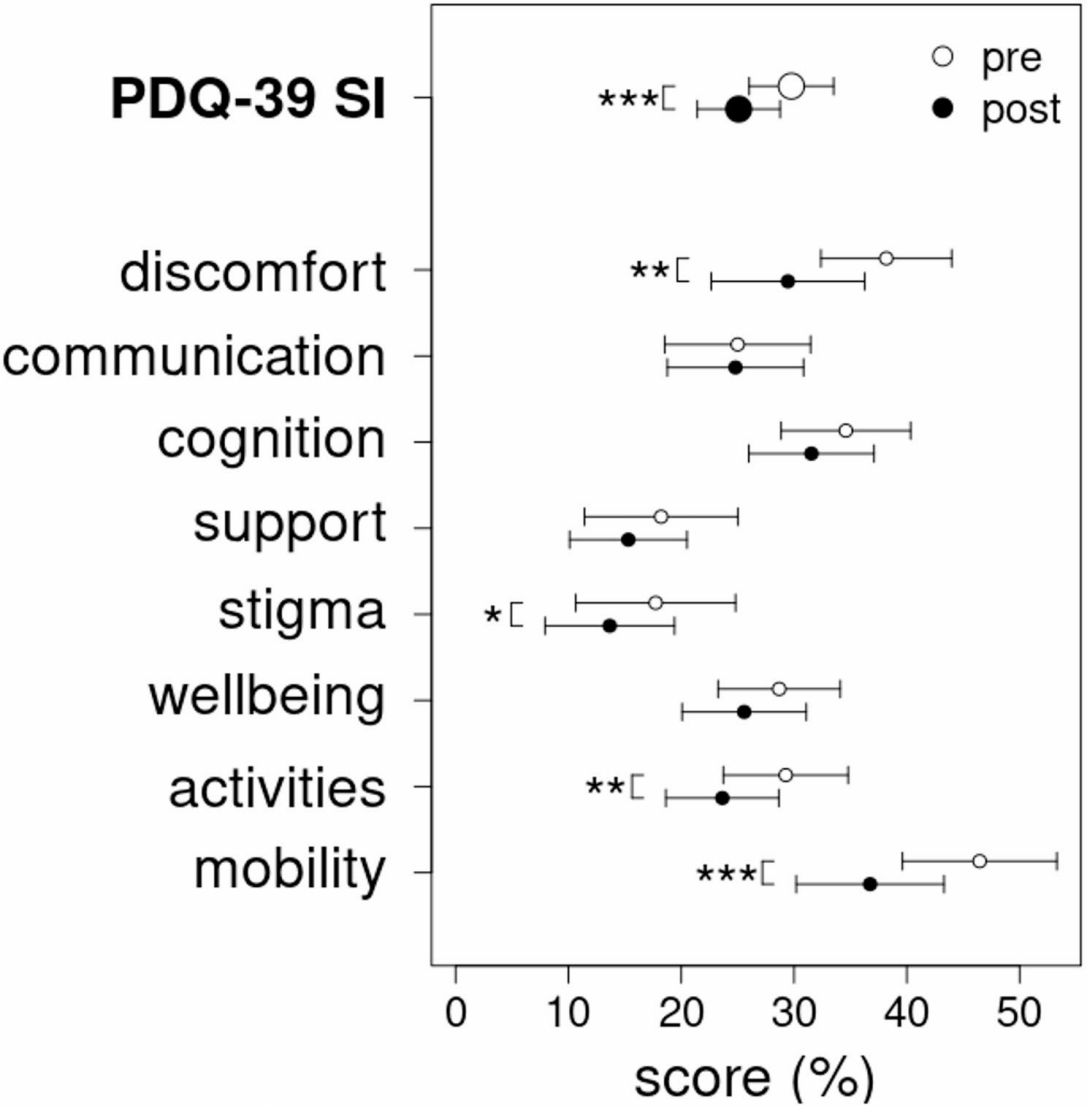
Changes in health-related quality of life. The total score of the health-related quality of life (PDQ-39 SI) and its subscores are illustrated as means before (solid circles) and after (open circles) the intervention. Errorbars are two standard errors of the mean value. Statistically significant differences are marked by asterisks (*p* < 0.05: *, *p* < 0.01: **, *p* < 0.001: ***)

Our analysis revealed a statistically significant improvement in the total PDQ-39SI score following PD-MCT. The magnitude of improvement was 4.672 points, representing a 15.7% reduction relative to the baseline pretest value. Significant improvements were also observed in the subscales of mobility, activities of daily living, stigma, and bodily discomfort (cf. Table 2; Fig. 2).

**Table 2:** Pre/post PD-MCT comparison of clinical scales and LEDD

	pre PD-MCT mean (sd)	post PD-MCT mean (sd)	*p*-value (statistic)^1^	effect size (*r*)
MDS-UPDRS part III	33.93 (15.51)	28.65 (13.02)	0.001 (V = 679)	0.496
TUG (s)	13.39 (5.58)	10.98 (4.59)	< 0.001 (V = 753.5)	0.583
PDQ39 SI	29.77 (12.29)	25.09 (12.03)	< 0.001 (V = 770)	0.547
- mobility	46.45 (22.44)	36.74 (21.48)	< 0.001 (V = 744.5)	0.621
- activities	29.26 (18.13)	23.64 (16.41)	0.003 (V = 573)	0.46
- emotional wellbeing	28.68 (17.75)	25.58 (18.04)	0.593 (V = 367.5)	-
- stigma	17.73 (23.22)	13.66 (18.81)	0.031 (V = 357)	0.373
- social support	18.22 (22.29)	15.31 (17.05)	0.455 (V = 163)	-
- cognition	34.59 (18.82)	31.54 (18.19)	0.124 (V = 498.5)	-
- communication	25.00 (21.21)	24.81 (19.79)	0.802 (V = 266)	-
- bodily discomfort	38.18 (19.01)	29.46 (22.3)	0.007 (V = 529)	0.447
LEDD	872.5 (485.06)	959.05 (555.59)	0.012 (V = 185.5)	0.401

^1^ Teststatistic V of Wilcoxon signed rank test

#### Custom questionnaire for patient satisfaction

**Fig. 3:**
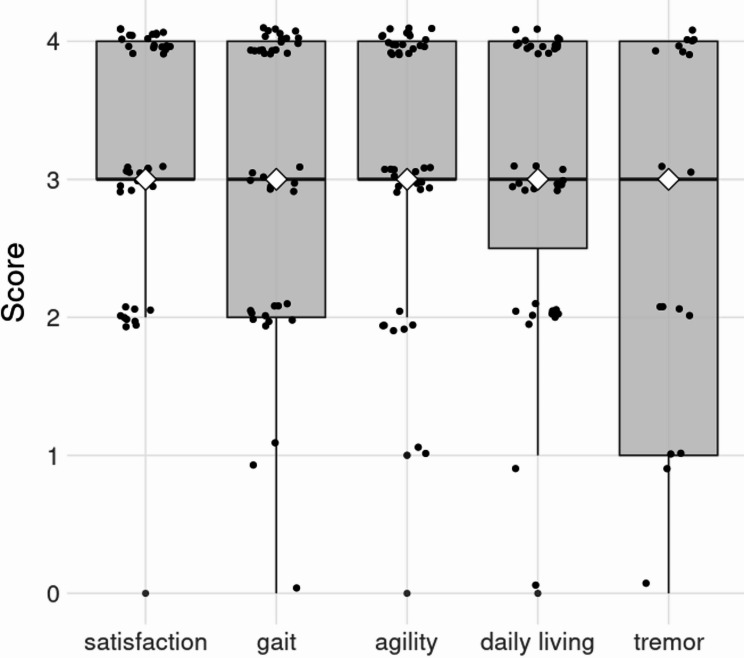
Boxplot with individual (black dots, randomly jittered for visibility) and median (white diamonds) responses to the self-report questionnaire for satisfaction with PD-MCT and subjective improvement in four different domains after the treatment

Self-reported satisfaction with and improvement after PD-MCT treatment was high across all assessed domains (see Fig. 3). The median satisfaction scores were all at three out of four possible points.

It is worth highlighting that the tremor domain exhibited greater variability in scores compared to other domains. This variability likely reflects the fact that tremor symptoms were present in only a subset of the patient sample, thereby influencing the overall results for this category.

#### Timed-up-and-go test (TUG)

The mean time to complete the TUG decreased statistically significant by 2.406 s of almost 18% between pre- and post-treatment measurements (cf. Table 2).

#### MDS-UPDRS part III

**Fig. 4:**
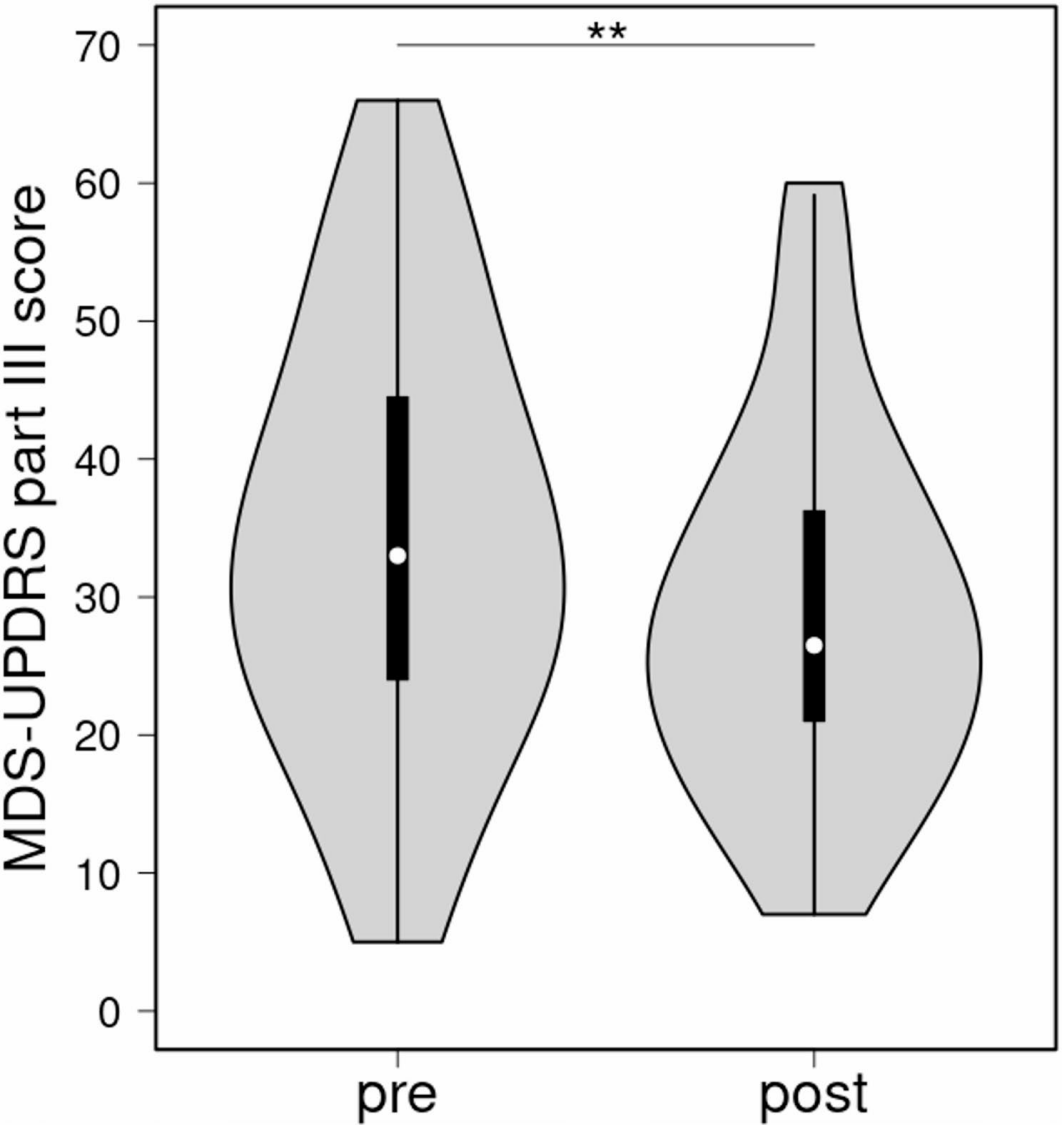
Violin of the MDS-UPDRS part III scores before and after treatment. The white dot represents the median, the black bar outlines the 25th -75th percentile. Statistical significance is marked by asterisks (*p* < 0.01: **)

Motor symptoms, as assessed by the MDS-UPDRS part III, showed significant improvement after the treatment period. The mean score decreased by 5.286 points (16%) from the pre-treatment mean score (cf. Table 2) A visual comparison of pre- and post-treatment scores is provided in Fig. 4.

### Gait analyses

**Fig. 5:**
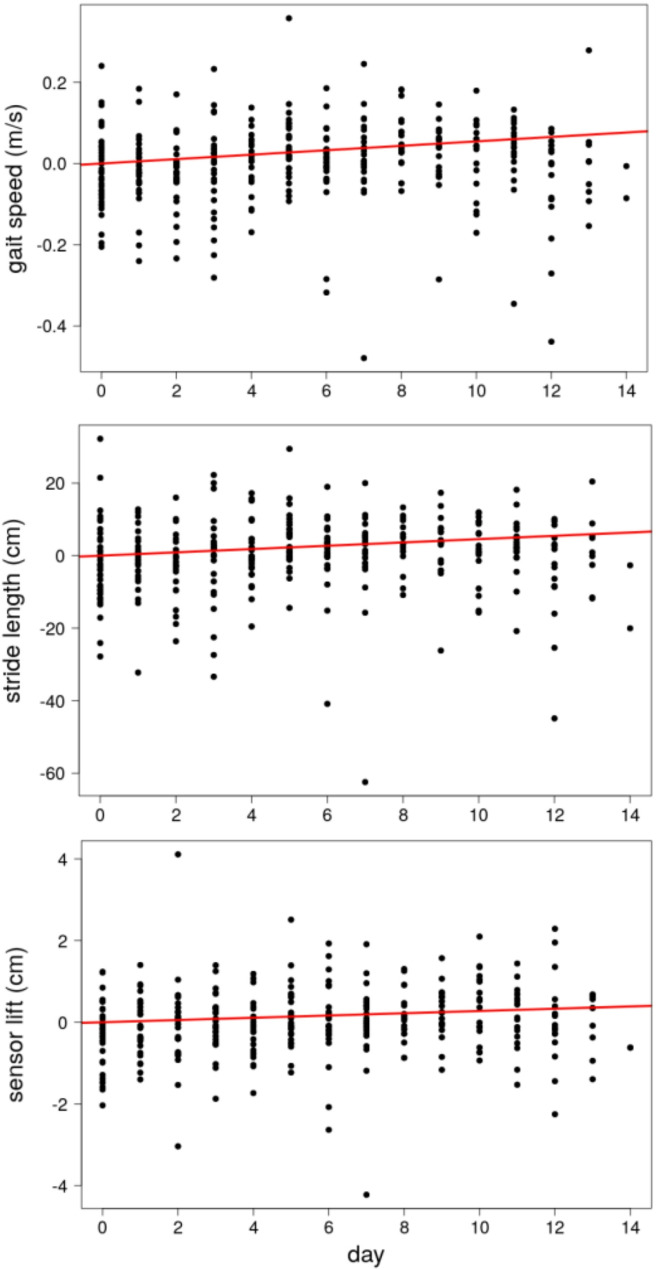
Results of the gait analysis over time. Per-patient averaged daily mean values for gait speed, stride length, and lift height, alongside fixed-effects predictions from linear mixed-effects models using treatment day as a predictor

Significant improvements were observed in all three gait parameters over the treatment period. Linear mixed-effects regression analysis, with patient identity included as a random factor, showed that gait speed increased by 7.54 cm/s (t = 66.44, *p* < 0.001). Stride length also increased by 6.4 cm over the course of the treatment (t = 62.67, *p* < 0.001). Furthermore, lift height showed a statistically significant improvement, with an increase of 3.86 mm over the same period (t = 28.16, *p* < 0.001). For a detailed visualization of the gait data and model predictions, please refer to Fig. 5.

### Other analyses

In our exploratory analyses of the effect of medication changes on outcome parameters, we found no significant Spearman rank correlations between pre/post-treatment differences in levodopa equivalent daily dose (LEDD) and the corresponding differences in PDQ-39 total scores, TUG test results, and MDS-UPDRS part III values (all *p* > 0.05). When incorporating LEDD as an additional predictor alongside treatment duration in a linear mixed effects model, statistically significant positive effects of LEDD on all three gait parameters were found. However, the magnitude of these effects was minimal.

For assessing demographics, we explored the influence of age, sex, Hoehn & Yahr stage as well as PD duration and DBS treatment group.

Patients treated with DBS reported higher satisfaction with PD-MCT scores compared to those without DBS. This trend was further supported in a subsample of 21 individuals (8 treated with DBS) who evaluated subjective tremor improvement, where DBS-treated patients again reported significantly higher improvement. Additionally, in the overall sample, male patients rated their subjective gait improvement higher than female patients.

Higher Hoehn & Yahr stages were significantly associated with greater pre/post-treatment improvements in both MDS-UPDRS part III scores and TUG test results. This relationship remained robust even when improvements were normalised by pre-treatment values, indicating that the observed effect is not merely attributable to a floor effect. Subjective treatment success, as measured by the “agility” scale, was positively correlated with objective improvements measured by slope coefficients of a per-individual linear regression of gait speed and stride length on treatment days. These findings suggest that perceived agility improvements align with measurable changes in mobility. A summary of all reported exploratory results is shown in supplementary Table 1.

## Discussion

In this study, we analysed 43 PD patients undergoing PD-MCT, a German healthcare programme tailored for patients suffering from PD. Notably, this is the first study to employ continuous sensor-based gait measurements throughout treatment, providing detailed, objective insights into motor function. Beyond gait, we examined factors influencing symptom improvement, offering fresh perspectives on the effectiveness of this comprehensive treatment approach.

Our findings highlight the significant benefits of PD-MCT across all measured outcomes. The PDQ-39 questionnaire showed a 15.7% improvement in quality of life post-treatment, exceeding the clinically significant threshold [37] and aligning with prior studies [24]. Improvements were most notable in mobility, activities of daily living, and bodily discomfort, likely due to targeted physical therapies. Interestingly, the stigma dimension also improved, possibly reflecting the normalising environment of the hospital. However, no significant changes were seen in cognition, social support, or communication, suggesting these areas may require longer-term interventions or enhancements to the PD-MCT programme. Importantly, improvements in PDQ-39 scores were unrelated to LEDD adjustments, underscoring the role of non-pharmacological interventions, such as physiotherapy and occupational therapy.

Patient-reported outcomes further demonstrated high satisfaction with PD-MCT. Subjective improvements in daily living, agility, gait, and tremor closely mirrored objective results. These were supported by a 16% improvement in MDS-UPDRS part III scores and an 18% improvement in TUG performance, aligning with prior studies [19, 20]. Our absolute improvement in UPDRS-III surpassed the clinically relevant threshold of 3.9 points [38], achieved within a relatively short time. Similarly, TUG results paralleled previous findings, reinforcing the value of non-pharmacological therapies in PD-MCT’s success.

For the first time, continuous sensor-based monitoring confirmed clinically meaningful improvements in gait parameters during treatment. Gait speed increased by 5.4 cm/s over 14 days, consistent with earlier findings [28]. Stride length and lift height also showed significant gains, with lift height being evaluated for the first time. While gait improvements correlated with LEDD changes, the effects were minor in size, highlighting the primary role of non-pharmacological interventions in driving these outcomes.

In summary, PD-MCT effectively improves symptoms across subjective, clinical, and objective measures. Post-hoc analyses revealed higher satisfaction among DBS-treated patients, particularly for tremor, and greater gait satisfaction in males, though the reasons remain unclear. Advanced disease stages showed larger clinical motor improvements, possibly due to earlier stages being adequately managed in outpatient settings. While no predictors of individual gait improvements were identified, gait changes correlated with perceived agility, suggesting a broader impact on lower-body mobility.

Our data emphasises the benefit of a multiprofessional approach to manage PD’s complex symptoms. This is evident in the independence of improvements from LEDD changes and the critical role of gait as a marker of quality of life [39–41]. Improving gait must remain a priority in PD treatment. Continuous monitoring, such as IMU-based assessments, offers an accessible and scalable solution for tracking gait and motor symptoms. Our study confirms the feasibility of integrating these technologies in hospital settings, complementing previous outpatient-focused research [41–44].

This study’s design as a single-centre cohort study imposes limitations. The lack of a control group increases the risk of bias from uncontrolled confounding variables or biased patient selection. Additionally, the single-centre design may limit generalisability, particularly given the relatively high proportion of DBS-treated patients in our sample. Furthermore, while we assessed the influence of LEDD in our analyses, we could not explore the effects of DBS adjustments, as patients could change stimulation programs and amplitude independently and exact timing of such changes was unavailable. Finally, testing multiple outcome parameters increases the risk of type I errors. For future research, we recommend that multicentre studies and randomised controlled trials be conducted to reduce bias and enhance generalisability. Such studies should also quantify non-pharmocological therapies in order to assess their effects. Another suggestion would be that analyses of insurance claims could be undertaken to measure the effect of PD-MCT on e.g. falls or re-hospitalisation.

## Conclusions

PD-MCT is a highly effective treatment within the German healthcare system, significantly improving multiple symptom domains, especially gait, for PD patients. Its benefits extend beyond medication adjustments, highlighting the value of non-pharmacological therapies. The programme’s comprehensive, multidisciplinary nature, in our opinion, justifies its cost and underscores the importance of holistic care in managing this complex disease. Based on our findings, we feel that multimodal treatment models should be more widely implemented and made more accessible within routine care to further enhance patient outcomes and quality of life.

## Electronic supplementary material

Below is the link to the electronic supplementary material.

Supplementary Material 1

## Data Availability

The code used for the statistical analysis is available at: https://github.com/kleinholdermann/pd_gait_pkb Pseudonymised data are available from the authors upon reasonable request.
