# Supplementary material for: Multidisciplinary inpatient care for Parkinson’s disease: a single-centre cohort study on improvements in gait, overall motor function, and quality of life
Source: Neurol Res Pract. 2025 Sep 2;7(1):62. doi: 10.1186/s42466-025-00422-y (PMC12406477; doi:10.1186/s42466-025-00422-y)
Supplement: Supplementary file 1 — Supplementary Material 1 [file 42466_2025_422_MOESM1_ESM.docx]

| N | 43 (21 female) | |
| --- | --- | --- |
| age  *mean (sd)* | 65.33 (8.62) | |
| Hoehn & Yahr stage  *median (IQR)* | 2 (1) | |
| H/Y 1 | 3 | |
| H/Y 2 | 24 | |
| H/Y 3 | 14 | |
| H/Y 4 | 2 | |
| PD duration in years  *mean (sd)* | 10.42 (7.04) | |
| patients treated with DBS | 18 | |
| patients using walking aids |  | |
| - walker | 6 | |
| - stick | 3 | |
| - crutches | 1 | |
|  | **pre-MCT** | **post-MCT**  *mean (sd)* |
| LEDD *(mean (sd)* | 872.5 (485.06) | 959.05 (555.59) |
| UPDRS part III *(mean (sd)* | 33.93 (15.51) | 28.65 (13.02) |
| TUG (s) *(mean (sd)* | 13.39 (5.58) | 10.98 (4.59) |
| PDQ39 SI *(mean (sd)* | 29.77 (12.29) | 25.09 (12.03) |
| - mobility *(mean (sd)* | 46.45 (22.44) | 36.74 (21.48) |
| - activities *(mean (sd)* | 29.26 (18.13) | 23.64 (16.41) |
| - emotional wellbeing *(mean (sd)* | 28.68 (17.75) | 25.58 (18.04) |
| - stigma *(mean (sd)* | 17.73 (23.22) | 13.66 (18.81) |
| - social support *(mean (sd)* | 18.22 (22.29) | 15.31 (17.05) |
| - cognition *(mean (sd)* | 34.59 (18.82) | 31.54 (18.19) |
| - communication *(mean (sd)* | 25.00 (21.21) | 24.81 (19.79) |
| - bodily discomfort *(mean (sd)* | 38.18 (19.01) | 29.46 (22.3) |

Supplementary Table 1. Results of exploratory analyses
